# Auditory-motor synchronization with temporally fluctuating sequences is dependent on fractal structure but not musical expertise

**DOI:** 10.3389/fpsyg.2014.00970

**Published:** 2014-09-03

**Authors:** Summer K. Rankin, Charles J. Limb

**Affiliations:** ^1^Sound and Music Perception Laboratory, Department of Otolaryngology Head and Neck Surgery, Johns Hopkins University School of MedicineBaltimore, MD, USA; ^2^Peabody Conservatory of the Johns Hopkins UniversityBaltimore, MD, USA

**Keywords:** 1/f, fractal, perception-action, synchronization, music perception, coordination, power law scaling, metastable

## Abstract

Fractal structure is a ubiquitous property found in nature and biology, and has been observed in processes at different levels of organization, including rhythmic behavior and musical structure. A temporal process is characterized as fractal when serial long-term correlations and statistical self-similarity (scaling) are present. Previous studies of sensorimotor synchronization using isochronous (non-fractal) stimuli show that participants' errors exhibit persistent structure (positive long-term correlations), while their inter-tap intervals (ITIs) exhibit anti-persistent structure (negative long-term correlations). Auditory-motor synchronization has not been investigated with anti-persistent stimuli. In the current study, we systematically investigated whether the fractal structure of auditory rhythms was reflected in the responses of participants who were asked to coordinate their taps with each event. We asked musicians and non-musicians to tap with 12 different rhythms that ranged from anti-persistent to persistent. The scaling exponents of the ITIs were strongly correlated with the scaling exponents of the stimuli, showing that the long-term structure of the participants' taps scaled with the long-term structure of the stimuli. Surprisingly, the performance of the musicians was not significantly better than that of the non-musicians. Our results imply that humans are able to readily adapt (rather than simply react) to the overall statistical structure of temporally fluctuating stimuli, regardless of musical skill.

## 1. Introduction

Many aspects of life require us to coordinate our behavior with the environment and with other people. Auditory-motor coordination plays a vital role in everyday activities, such as speech, and also in more abstract pursuits like music and dance. Understanding the underlying processes that allow us to accomplish such complex coordination tasks is vital to understanding both normal and abnormal brain function. Many studies have used periodic motor behavior to probe the perception of time and the ability to coordinate with external stimuli (reviewed by Repp, 2005; Repp and Su, 2013). Using different auditory-motor synchronization paradigms, some of these studies have found fractal structure in various aspects of human rhythmic and coordinated motor behavior (Gilden et al., 1995; Chen et al., 1997; Madison, 2001, 2004; Chen et al., 2002; Delignières et al., 2004; Lemoine et al., 2006; Rankin et al., 2009). There have been few studies on synchronization with fractal stimuli. A handful of studies have investigated stride intervals cued by fractal auditory (Hove et al., 2012; Kaipust et al., 2013; Marmelat et al., 2014) and visual (Rhea et al., 2014) stimuli. Stephen et al. (2008) asked participants to tap with a chaotic visual signal, but sensorimotor synchronization has not been systematically investigated with auditory stimuli that are fractal in nature.

Fractal, or 1/*f*, time-series exhibit long-term memory that can be positively (*persistent*) or negatively (*anti-persistent*) correlated. In a persistent time-series, large intervals (between events) are likely to be followed by large intervals, while small intervals by other small intervals. In an anti-persisent time-series, large intervals are likely to be followed by small intervals while small intervals are likely to be followed by large intervals. These long-term correlations imply that a fractal time-series is strongly dependent on its own history. This property is referred to as the long-term memory of a fractal time-series. Fractal structure has been observed in natural behavior such as healthy gait (Delignières et al., 2009), the production of periodic movements (Michon, 1967; Chen et al., 1997; Madison, 2001, 2004), and self-paced movements (Lemoine et al., 2006). Chen et al. (1997) suggest that the long-range dependence of timing fluctuations is the outcome of distributed neural processes acting on multiple time scales. In addition, fractal structure is considered a hallmark of *metastability* (Friston, 1997; Freeman and Holmes, 2005; Werner, 2010; Tognoli and Kelso, 2014) in the brain. Metastability is the ability of neural systems to integrate functionally segregated entities (e.g., brain areas, end effectors) in a task relevant manner in space and time. Thus, it is an indicator of our ability to adapt and coordinate to the external world. In this paper we investigated auditory-motor synchronization in musicians and non-musicians using anti-persistent, uncorrelated, and persistent auditory stimuli.

Most studies that investigate motor coordination measure self-paced motor behavior or ask participants to synchronize with an isochronous stimulus. In self-paced continuation tapping studies, participants are presented with an isochronous auditory rhythm and asked to tap in synchrony with each event and continue tapping at the same rate after the stimulus has stopped. Several studies have shown that the variability in the inter-tap intervals (ITIs) follows a fractal structure that is persistent (Gilden et al., 1995; Chen et al., 2002; Delignières et al., 2004; Madison, 2004; Lemoine et al., 2006). In other words, while the continuation taps matched the period of the stimuli, the fluctuations in the ITIs exhibited fractal structure. Conversely, when synchronizing with an isochronous stimulus, participants' asynchronies (error time-series) exhibited persistence (Chen et al., 1997, 2002; Pressing and Jolley-Rogers, 1997), while ITIs exhibited anti-persistence (Chen et al., 1997, 2002).

Relatively few studies have investigated entrainment with temporally fluctuating stimuli (Thaut et al., 1998, 2009; Drake et al., 2000; Dixon et al., 2006). When fluctuations are small, participants track the changes and tap exactly one event late (Thaut et al., 1998). This results in a high cross-correlation of the ITIs with inter-onset intervals (IOIs) at lag one. However, when given a piece of music containing large fractal tempo fluctuations, participants successfully synchronized with the beat (Repp, 2002; Rankin et al., 2009) which resulted in a strong cross-correlation between ITIs and inter-beat intervals at lag zero. This indicates that participants were predicting (rather than tracking) the next onset in order to coordinate their motor behavior with each event. Furthermore, the 1/*f* structure of the stimulus was reflected in the participants' ITIs. Stepp and Turvey (2009) propose that when a stimulus contains temporal fluctuations, people will coordinate on a non-local time scale, referred to as *strong anticipation*. It is likely that in order to adapt to a fluctuating stimulus, participants are doing a mixture of tracking and predicting, which leads to structural similarity in the statistics of the ITIs results in making the statistical structure of the ITIs similar to the stimulus. This adaptation to the statistical structure of the stimulus can be measured by comparing the long-range correlations of the behavior to the long-range correlations of a stimulus.

While previous literature has explored the persistent range of stimuli, no studies have systematically investigated sensorimotor synchronization with the stimuli ranging from anti-persistent to persistent. Here we investigated the extent to which participants (musicians and non-musicians) could adapt to the statistical structure of fluctuating auditory stimuli. Participants were asked to tap in synchrony with each event of a fractal rhythm that varied from anti-persistent to persistent. Under this paradigm we tested two hypotheses. The first hypothesis was that the long-term statistical structure of the auditory stimuli would be reflected in the behavior of all subjects. If verified, the fractal exponents obtained from the inter-tap intervals would be strongly correlated with the fractal exponents of the stimuli. In other words, as the structure of the stimuli varied from anti-persistent to persistent the structure of the taps would co-vary.

The second hypothesis was that the auditory-motor synchronization of musicians would be better than that of the non-musicians. Previous studies have shown that tempo fluctuations in musical performances contain persistent structure (Rankin et al., 2009). Music gives many cues that help the listener predict upcoming tempo fluctuations: rhythm, harmonics, dynamics, etc. In this paper, we used persistent stimuli with fluctuations that were similar to music but contained no musical or other information about when an upcoming event would occur. Since musicians have extensive experience coordinating with other people who are fluctuating in a (presumably) fractal manner and have lower variability when synchronizing with isochronous external stimuli and have more extreme lower and upper rate limits than non-musicians (Repp, 2005), we hypothesized that the musicians would be better able to coordinate with the stimuli, in comparison to non-musicians.

## 2. Materials and methods

### 2.1. Stimuli

We created 12 discrete log-normally distributed time-series (512 points) which contained systematically varying amounts of structure (β = −1.21, −1.03, −0.83, −0.60, −0.40, −0.20, 0.00, 0.19, 0.41, 0.59, 0.98, 1.21) and an isochronous control sequence (IOI = 563.3 ms). The 12 time-series, each with a specific value of β, were created using the spectral synthesis method (Turcotte, 1997).

The first 262 values of each sequence were treated as inter-onset intervals (IOIs; the mean IOI = 562.3 ± 165.7 ms was chosen because it is within the range of maximum sensitivity–approximately 600 ms–for tempo and interval discrimination (Drake and Botte, 1993) and were cumulatively summed to create the stimuli (available in Supplementary Materials). Each stimulus consisted of 261 Hz pure tones that were 150 ms in duration (approximately middle C on a piano). Analyses were conducted and stimuli were created using MATLAB (The MathWorks, Inc, Natick, MA).

**Figure 1:**
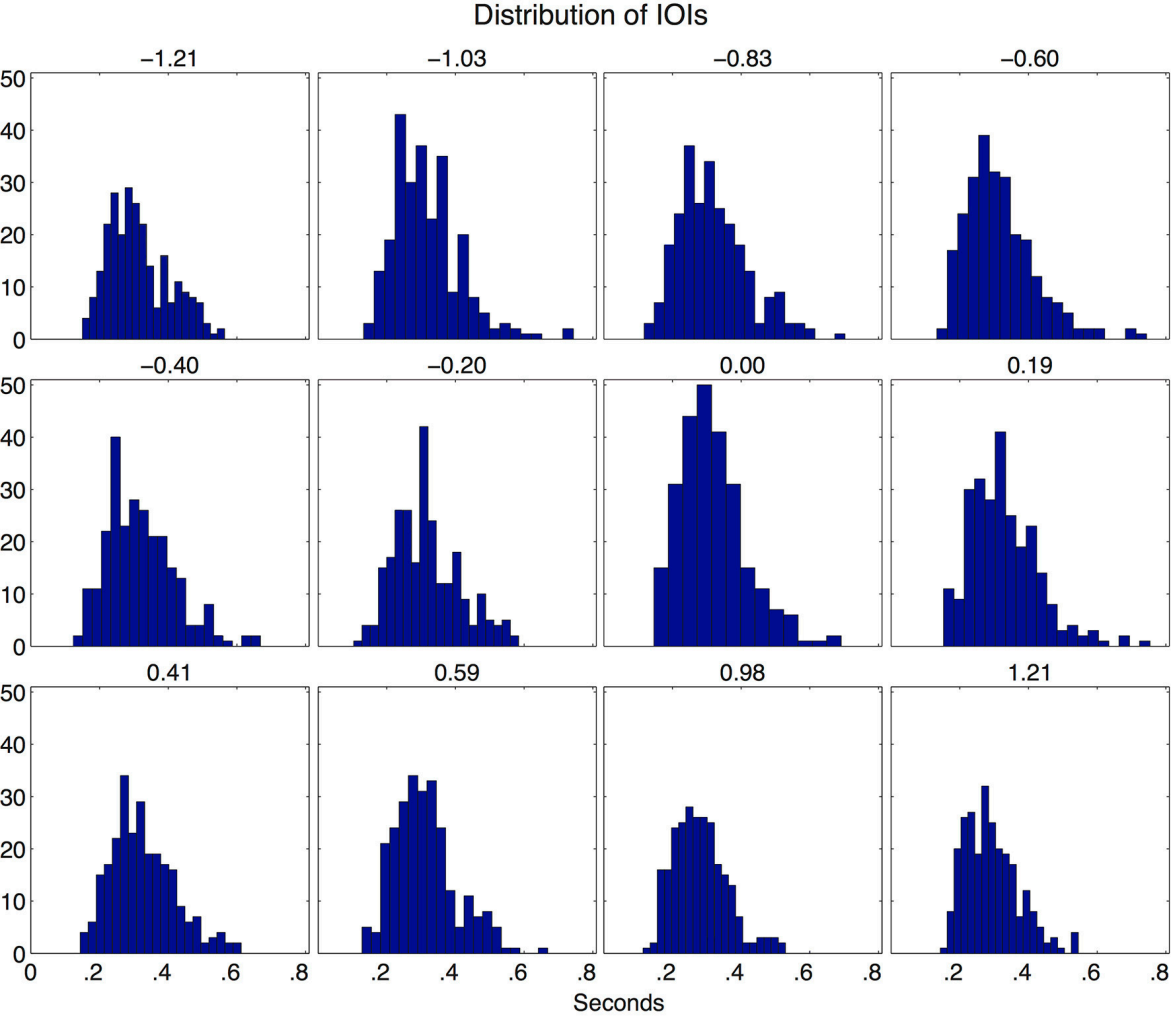
**The log-normal distribution for each non-isochronous stimulus is shown in a series of histograms**.

We created the self-affine log-normally distributed 1/*f* noise as follows:

1. A pseudorandom random time-series, *y_n_*, *n* = 1, 2, 3, …, *N*, was created with length *N* = 512, and auto-correlation at lag 1, *ac*_1_ = 0.00.
2. This time-series was transformed to have a normal Gaussian distribution using the relation
  (1) $$y_{n1}=\frac{\log(y_{n})-\mu (\log(y_{n}))}{\sigma (\log(y_{n}))}$$
  where the total time interval, *T*, has been divided into *N* equal intervals of length, δ, $\delta =\frac{T}{N}$. The units of δ are those of *T*; *N* is dimensionless.
3. A Discrete Fourier Transform was taken of the time-series, *y*_*n*1_. The Fourier coefficients are given by
  (2) $$Y_{m}=\delta \sum_{n\;=\;1}^{N}y_{n1}e^{2\pi inm/N},\;m=1,2,3,\ldots ,N$$
  This transform maps *N* real numbers (*y_n_*) into *N* complex numbers (*Y_m_*). The Fourier spectrum of white noise will be flat, β = 0. Except for the statistical scatter, the amplitudes of |*Y_m_*| will be equal.
4. The resulting Fourier coefficients, *Y_m_*, were filtered using the relation
  (3) $$Y_{m}^{\prime }={\left(\frac{m}{N}\right)}^{-\beta /2}Y_{m}$$
  The power β /2 is used because the power spectral density is proportional to the amplitude squared. The amplitudes of the small-*m* coefficients correspond to short wavelengths, and high frequencies. The large-*m* coefficients correspond to long wavelengths and low frequencies.
5. An Inverse Discrete Fourier Transform (IDFT) was taken of the filtered Fourier coefficients. The sequence of points is given by
  (4) $$X_{n}=\frac{1}{N\delta }\sum_{m\;=\;1}^{N}Y_{m}^{\prime }e^{-2\pi inm/N},\;m=1,2,3,\ldots ,N$$
  These points constitute the fractional Gaussian noise.
6. Many naturally occurring time-series have only positive values, which results in a non-Gaussian distribution (Turcotte, 1997; Malamud and Turcotte, 1999). Previous results have shown that musical tempi (collected from performance data) exhibit a lognormal distribution (Rankin et al., 2009). Thus, the fractional Gaussian noise sequence was converted into a fractional log-normal noise sequence using the relation -
  (5) $$\begin{array}{l} \;Z=\exp\left(\frac{X_{n}-\log\left(\mu /\sqrt{1+c_{v}^{2}}\right)}{\sqrt{\log(1+c_{v}^{2})}}\right),\\ m=1,2,3,\ldots ,N \end{array}$$
  The resultant time-series represents a realization of log-normal distributed 1/*f* noise (Figure 1) with β representing the strength of long-range persistence or anti-persistence, mean value μ = 0.3178 and coefficient of variation *c_v_* = σ/μ = 0.308. This *c_v_* was selected to be similar to the *c_v_* values from previous studies of tempo fluctuation (Rankin et al., 2009). This method was used to create the time points for the 12 non-isochronous stimuli.

### 2.2. Participants

We recruited a group of non-musicians (*n* = 8) and a group of musicians (*n* = 8) from the Johns Hopkins University community; a total of 16 volunteers (10 males, 6 females, mean age = 26.12 ± 5.77 years, age range = 19–37 years) participated in the study. The study was approved by the Institutional Review Board at Johns Hopkins University School of Medicine. All participants gave written informed consent and were compensated for their participation. By self-report, all participants had normal hearing and had no known neurological problems. The amount of musical training for the musician group ranged from 10 to 27 years, average years of formal music lessons = 16.75 ± 5.70). Participants played a variety of instruments: 1 violin/guitar/voice, 2 voice, 1 sax, 1 cello/piano, 1 cello, 1 trombone, 1 vocal percussion.

### 2.3. Procedure

Participants were instructed to tap along with each sound (event) and to keep up with or anticipate the tempo changes to the best of their ability as though tapping along with a piece of music. Participants tapped with the index finger of their dominant hand on the “1” key of a standard wireless Dell computer keyboard. There was no auditory feedback from the taps. Participants were seated in a sound isolation booth, and auditory sequences were presented via a single calibrated loudspeaker with the volume adjusted to a comfortable level for each individual. Each of the 13 stimuli were presented once, in a pseudorandom order. The experiment was approximately 45 min in duration. E-Prime 2.0 Professional software (Psychology Software Tools, Pittsburgh, PA) was used to present the stimuli and collect the data.

### 2.4. Analysis

The stimuli (IOIs) and the collected data (ITIs) were analyzed using a power spectral density (PSD), and rescaled range (R/S) analysis (Bassingthwaighte et al., 1994; Feder, 1988; Witt and Malamud, 2013). Fractal time-series exhibit statistical self-similarity given by the power-law *S*(*f*) ~ 1/*f*^β^, where the spectral density, *S*(*f*), scales with frequency, *f*, as a function of β (Bassingthwaighte et al., 1994). The exponent β determines the slope of the power-law behavior which describes the nature of the long-term memory. Most naturally occurring 1/*f* series are characterized by positive long-term correlations (β > 0), or persistence. According to Rangarajan and Ding (2000), simply having a straight line on a log-log plot of a power spectrum does not necessarily mean that something contains fractal structure. Especially when dealing with time-series, one must look at the long-term correlation using additional other analyses, such as Hurst's Rescaled Range (R/S) analysis.

The PSD was calculated for the ITI time-series for each trial (β_*ITI*_). The first 6 taps of each trial were discarded and the remaining responses were used for analyses (see Table 1). The results of the PSD revealed that for each trial β_*ITI*_ < 1, therefore all trials were classified as stationary fractional Gaussian noise *fGn* and the R/S analysis was used (Eke et al., 2000, 2002) to confirm the results from the PSD (Rangarajan and Ding, 2000). In general, the *H_ITI_* (R/S) and β_*ITI*_ values agreed according to the equation β = 2*H* − 1.

**Table 1:** **Number of taps per condition (columns = β value)**.

	**Isoch**.	**−1.21**	**−1.03**	**−0.83**	**−0.60**	**−0.40**	**−0.20**	**0.00**	**0.19**	**0.41**	**0.59**	**0.98**	**1.21**
Mean	253	267	272	272	272	258	272	273	272	263	271	260	266
*SD*	4.37	19.38	15.43	16.14	15.79	21.02	15.96	16.77	16.05	17.15	15.26	12.37	28.29

Each stimulus contained 256 events (the first 6 taps and stimulus events have been removed).

**Figure 2:**
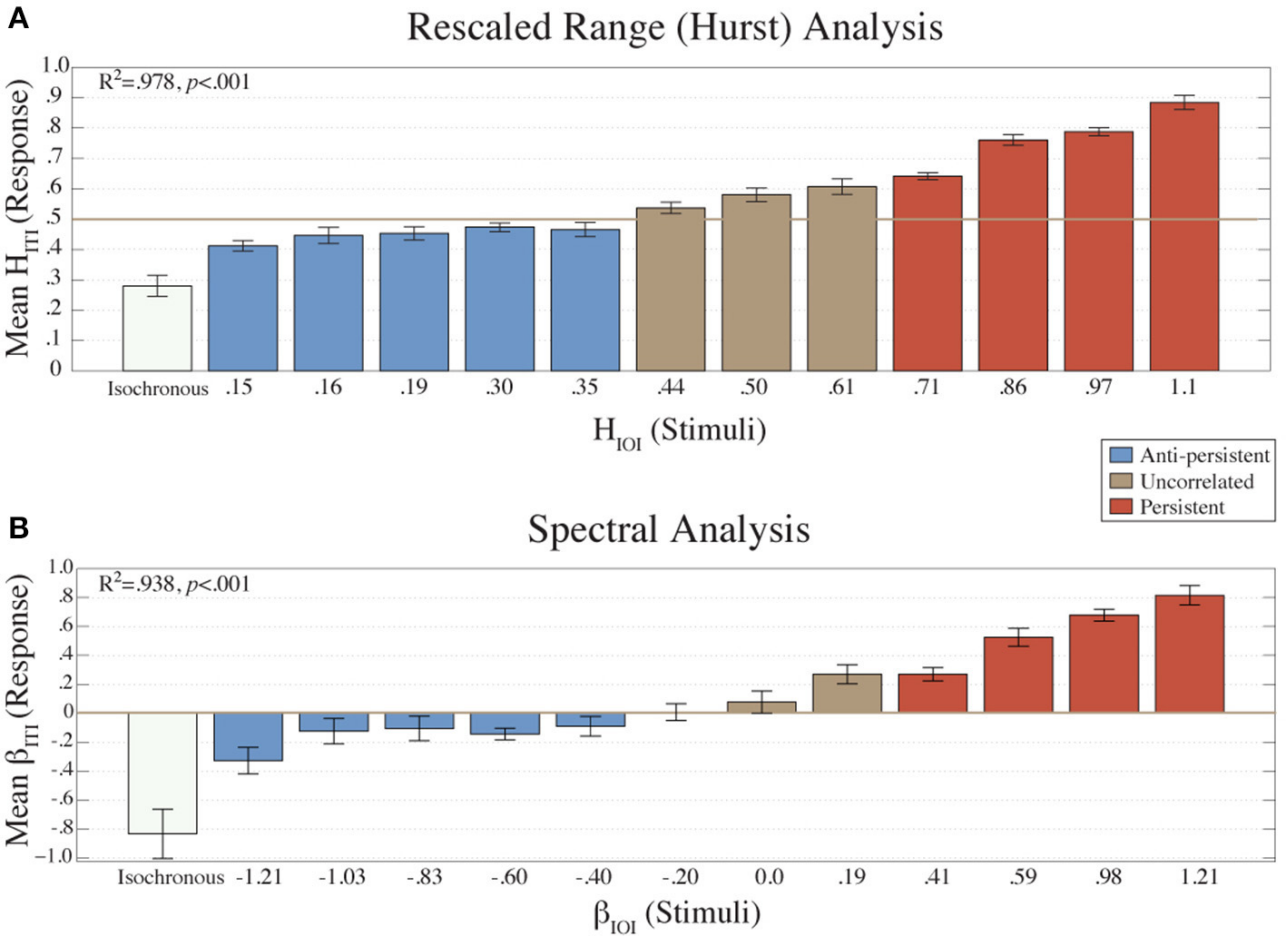
**The mean *H_ITI_* values (*N* = 16) from the R/S analysis are plotted (A) for each condition (*H_IOI_*)**. The mean β_*ITI*_ values (*n* = 16) from the Power Spectral Density analysis are plotted **(B)** for each condition (β_*IOI*_). Error bars represent the standard error of the mean. The colors represent the amount of long-term correlation that the stimulus contained.

The R/S analysis yields a Hurst exponent *H*, which gives a measure of smoothness of the time-series. *H* can assume any value between zero and one. A low *H* indicates a high degree of roughness or anti-persistence, and a high *H* indicates smoothness or persistence. A high *H* means that the object almost fills the next dimension, and *H* = 0.5 indicates no correlation in the series (white noise) (Hurst, 1951; Feder, 1988). When *H* equals 0.5 there is no long-term correlation present in the series. Persistence, 0.5 < *H* < 1, indicates positive long-term correlation. Anti-persistence, 0 < *H* < 0.5, indicates negative long-term correlation. Though this classification is theoretically valid, in the literature the boundaries between persistent, and antiperistent states are more broadly defined (Delignières et al., 2006). Based on this literature we defined the time-series as; persistent when *H* ≥ 0.7; β ≥ 0.4; uncorrelated when 0.4 ≤ *H* < 0.7; −0.2 ≤ β < 0.4; and anti-persistent when *H* < 0.4; β < −0.2 (see Figure 2).

In addition to the fractal analyses, we calculated the asynchrony between the stimulus and response. Asynchrony is a measure of error and is defined as the time between the stimulus onset (OT) and the time of the corresponding response (TT) thus; *asy_i_* = *TT_i_* − *OT_i_*.

## 3. Results

We confirmed that the statistics from the PSD and R/S analysis converged to ensure that the estimates of the fractal structure were accurate (Rangarajan and Ding, 2000).

The mean β_*ITI*_ values from each condition (Figure 2B) were fitted to the β values of the stimuli (β_*IOI*_) using a linear regression model. There was a strong, positive correlation (*R*^2^ = 0.938, *p* < 0.001) between the two.

Similarly, the mean *H_ITI_* values (Figure 2A) fitted to the *H_IOI_* values with a linear regression model also showed a high positive correlation (*R*^2^ = 0.978, *p* < 0.001). These results indicate that the structure of the ITIs was dependent on the structure of the stimulus.

A one-way ANOVA was calculated on the β_*ITI*_ and *H_ITI_* exponents with the factor Condition (β_*IOI*_). This revealed a main effect of condition [for β: *F*_(12, 195)_ = 13.03, *p* < 0.001; for *H*: *F*_(12, 195)_ = 61.99, *p* < 0.001].

**Figure 3:**
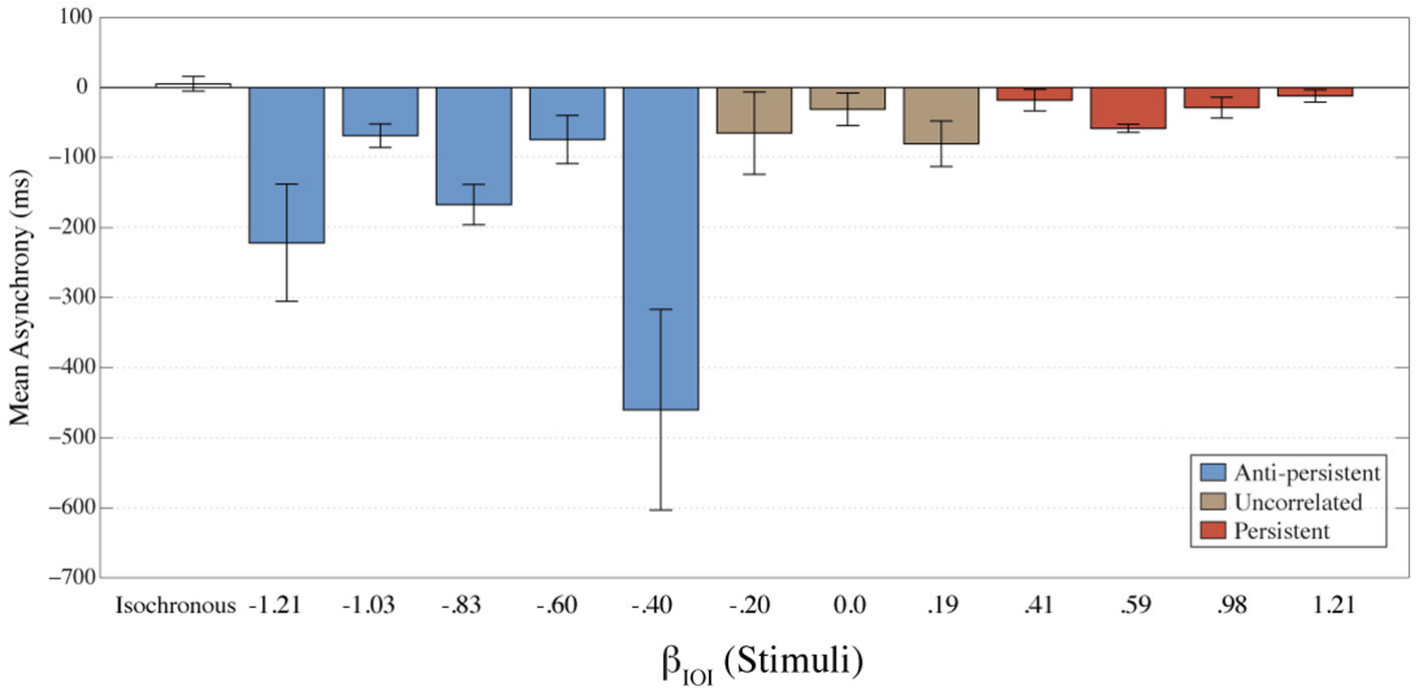
**The mean asynchrony for each condition is shown here**. Error bars represent the standard error of the mean. The colors represent the amount of long-term correlation that the stimulus contained.

**Figure 4:**
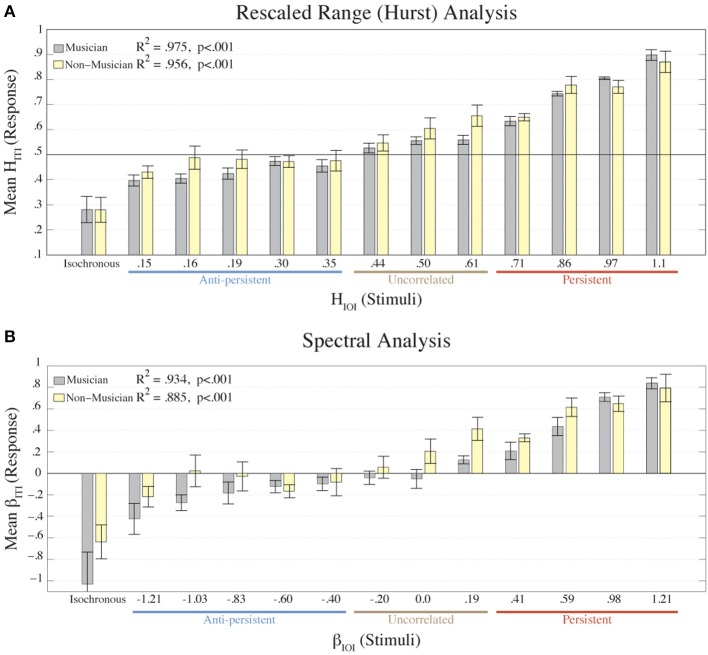
**The mean *H_ITI_* values of each group (musician, *n* = 8; non-musician *n* = 8) from the R/S analysis are plotted (A) for each condition (*H_IOI_*)**. The mean β_*ITI*_ values of each group (musician, non-musician) from the Power Spectral Density analysis are plotted **(B)** for each condition (β_*IOI*_). Error bars represent the standard error of the mean. The colors represent the participant groups: musicians = gray; non-musicians = yellow. The results from the linear regression of the mean ITIs fitted to the IOIs (excluding the isochronous condition) are shown in each plot. Below the X-axis we denote the amount of correlation that the stimulus contained.

The results of the fractal and error analyses presented in Figures 2–**4** are organized by separating the fractal structure of the stimuli into the following categories: anti-persistent, uncorrelated, and persistent. For subsequent analyses the values from the conditions within a category were collapsed together. A *t*-test revealed that *H* and β values for each category were significantly different (*p* < 0.05). The correlation of the *H_ITI_* against *H_IOI_* was also calculated for all categories. For anti-persistent, uncorrelated, and persistent categories, the *R*^2^ values were 0.7846, 0.9542, and 0.9862, respectively. Similarly, the *R*^2^ values for β_*ITI*_ and β_*IOI*_, were 0.7185, 0.9609, and 0.9666, respectively.

Asynchronies between the response and stimulus were calculated to determine the temporal relationship between the stimulus and responses. Asynchrony is considered to be a measure of the errors. A negative asynchrony indicates that a tap occurred before the stimulus; a positive asynchrony indicates that the tap occurred after the stimulus; and when asynchrony is zero it indicates that the tap occurred at exactly the same time as the stimulus. The mean asynchrony for each condition is shown in Figure 3. For all fractal conditions mean asynchrony was negative. This indicates that participants were tapping ahead of the stimuli. These results also suggest better synchronization (smaller asynchrony) as the stimulus gets more persistent. The isochronous condition had the smallest mean asynchrony.

Figure 4 shows the mean β_*ITI*_ and *H_ITI_* values from each condition for musicians and non-musicians. Using a linear regression model, the results for the musicians were the following: β_*ITI*_ fitted to β_*IOI*_, was *R*^2^ = 0.934, *p* < 0.001, and *H_ITI_* fitted to *H_IOI_* was *R*^2^ = 0.975, *p* < 0.001. The results for the non-musicians were: β_*ITI*_ fitted to β_*IOI*_ was *R*^2^ = 0.885, *p* < 0.001, and *H_ITI_* fitted to *H_IOI_* was *R*^2^ = 0.956, *p* < 0.001. Though the musicians had a stronger correlation between ITIs and IOIs than the non-musicians, the two groups were not significantly different according to two-way ANOVAs on the β_*ITI*_ and *H_ITI_* values with factors (Condition × Participant Type) (for β: *p* = 0.237; for *H*: *p* = 0.0446). A two-way ANOVA was also calculated on the asynchronies with factors (Condition × Participant Type). A main effect of Condition was revealed [*F*_(1, 12)_ = 3.47, *p* = 0.001], but no significant difference was found between musicians and non-musicians (*p* = 0.086). These results directly refute our hypothesis that musical expertise would lead to better synchronization with fractal stimuli.

## 4. Discussion

In this paper we tested two hypotheses regarding auditory-motor synchronization in humans. We found evidence to support one of these hypotheses while the other was not confirmed. As hypothesized, persistent stimuli yielded persistent ITI time-series, while uncorrelated and anti-persistent stimuli yielded uncorrelated ITIs. We found that systematic changes in the statistical structure of an auditory stimulus were reflected in the participants' responses. Furthermore, *H_ITI_* and β_*ITI*_ values for each condition were correlated with the *H_IOI_* and β_*IOI*_ values.

The fact that the tapping data scaled with the stimuli illustrates the participants' ability to predict upcoming fluctuations rather than to simply react to each event or track them at a lag of 1. If participants were exclusively tracking the stimuli, the structure of the ITIs would be smoother than the IOIs due to low-pass filtering of fluctuations (Dixon et al., 2006). The scaling of the behavior with the stimuli provides support for strong anticipation as the mechanism for prediction. Stepp and Turvey (2009) state that anticipating synchronization is an example of model-independent strong anticipation. Stephen et al. (2008) argue that an anticipatory system is not characterized by whether it is early or late for any one event. The system is anticipatory because of its dependence on future states. There is global coordination on a non-local time scale which can be measured by comparing the long-range correlations of the behavior and the long-range correlations of the system. The scaling exponent of the behavior should depend on the scaling parameter of the environment if strong anticipation is taking place, as shown by our data.

Though the general fractal nature of the stimuli is clearly reflected in the responses, there is a difference in the way in which participants respond to anti-persistent and persistent stimuli. The structure of the persistent stimuli was better represented in the participants' responses than the structure of the anti-persistent stimuli. This is evidenced by two measures. First, both *H_ITI_* and β_*ITI*_ values of persistent conditions were significantly different from the values of the anti-persistent conditions (*p* < 0.001). Second, when mean response values (mean *H_ITI_* and β_*ITI*_) are correlated against the stimulus values (*H_IOI_* and β_*IOI*_), the persistent conditions yield a higher correlation than the anti-persistent conditions. The isochronous condition resulted in strong anti-persistence for the ITIs and persistence for the error time-series (asynchronies). This finding is in agreement with previous studies of isochronous synchronization (Chen et al., 2002). Grigolini et al. (2009) have hypothesized that as a task gets harder, the spectral slope gets closer to β = 0 (less correlated). These data suggest that the anti-persistent condition was more difficult for subjects.

We also hypothesized that musicians as a group would synchronize better than non-musicians. As music is an acoustically rich and complex phenomena in which entrainment plays a crucial role, trained musicians tend to perform better at synchronization tasks than non-musicians (Repp, 2005; Repp and Su, 2013). However, we did not find evidence to support this hypothesis fully. Though the mean responses of the musicians were slightly more correlated with the stimuli in comparison to the non-musicians, these differences were not statistically significant. Furthermore, the analysis of the asynchronies showed that all participants tended to anticipate (negative mean asynchrony) the stimulus rather than track. Further subdivision of the asynchronies into musicians and non-musicians did not show any significant differences. This suggests that both groups performed the task similarly. This surprising finding may be attributable to the fact that temporal sequences are relatively impoverished forms of stimuli in comparison to the real-word forms of music for which musicians have a performance advantage. This finding may also suggest that the motor requirements involved in this task are not skill dependent, but instead may involve a more fundamental form of motor behavior. Coordination is a learned behavior that is heavily influenced by the environment (Pecenka and Keller, 2011); thus, it is possible that learning to coordinate with fractal stimuli is part of our normal developmental process.

Fractal structure and our ability to coordinate with it may have profound implications on neural processing. Clinical studies have shown that fractal structure is found in normal physiologic movements and biological processes and that the fractal structure breaks down in a wide range of disease states (e.g., Peng et al., 1995; Hove et al., 2012; Marmelat and Delignières, 2012). The fact that the structure of the ITIs scales with the stimuli indicates that the underlying process is not random and furthermore suggests that the system that produced the behavior is metastable. This underlying metastability is likely to be critically important for one's ability to engage dynamically with the environment. In addition, the ability to adapt to fractal stimuli may have important implications for aesthetically motivated behaviors. In studies that involve the aesthetics of fractal structure in visual scenes (both natural landscapes and abstract art) Spehar et al. (2003) found that there is a preferred or optimal range of β. Our results show that auditory-motor synchronization with a temporally fluctuating stimulus is dependent on the long-term structure of the stimulus, rather than musical skill or training. These results may reflect the presence of an optimal range of β for aesthetic preferences with respect to complex auditory stimuli such as music. In other words, it is plausible that humans have evolved to process 1/*f* structure more efficiently and that, consequently, stimuli that are fractal in structure are perceived as more aesthetically pleasing. Further studies are needed to clarify the complex relationship between music and the neural processing of fractal structure.

## Funding

Funding was provided by the DANA Foundation and the Brain Sciences Institute of the Johns Hopkins University School of Medicine.

### Conflict of interest statement

The authors declare that the research was conducted in the absence of any commercial or financial relationships that could be construed as a potential conflict of interest.
